# GLP-1 Receptor Agonists and Blood Pressure: A State-of-the-Art Review of Mechanisms, Evidence, and Clinical Implications

**DOI:** 10.1093/ajh/hpaf205

**Published:** 2025-10-23

**Authors:** Areesha Moiz, Tetiana Zolotarova, Kristian B Filion, Mark J Eisenberg

**Affiliations:** Centre for Clinical Epidemiology, Lady Davis Institute, Jewish General Hospital/McGill University, Montreal, QC, Canada; Centre for Clinical Epidemiology, Lady Davis Institute, Jewish General Hospital/McGill University, Montreal, QC, Canada; Centre for Clinical Epidemiology, Lady Davis Institute, Jewish General Hospital/McGill University, Montreal, QC, Canada; Department of Epidemiology, Biostatistics and Occupational Health, McGill University, Montreal, QC, Canada; Department of Medicine, McGill University, Montreal, QC, Canada; Faculty of Medicine, McGill University, Montreal, QC, Canada; Centre for Clinical Epidemiology, Lady Davis Institute, Jewish General Hospital/McGill University, Montreal, QC, Canada; Department of Epidemiology, Biostatistics and Occupational Health, McGill University, Montreal, QC, Canada; Department of Medicine, McGill University, Montreal, QC, Canada; Faculty of Medicine, McGill University, Montreal, QC, Canada; Division of Cardiology, Jewish General Hospital/McGill University, Montreal, QC, Canada

**Keywords:** glucagon-like peptide-1 receptor agonists, blood pressure, hypertension, antihypertensive, cardiometabolic risk

## Abstract

**Background:**

Glucagon-like peptide-1 receptor agonists (GLP-1 RAs) are widely used for the treatment of type 2 diabetes and, more recently, for weight management among individuals without diabetes.

**Aim:**

This review synthesizes the current evidence on the mechanisms by which GLP-1 RAs affect BP, their clinical effects across populations, and the implications for patient care. We discuss subpopulations who may benefit from their BP-lowering effects, identify limitations in the existing evidence, and explore future directions for research.

**Results:**

Beyond their metabolic effects, growing evidence suggests that GLP-1 RAs produce modest reductions in BP, typically 2-5 mm Hg systolic, across diverse populations with diabetes, obesity, or at high cardiovascular risk. These reductions appear to be driven primarily by weight loss, with additional contributions from potential weight-independent mechanisms such as natriuresis, improved endothelial function, and attenuation of vascular inflammation. Although smaller in magnitude than those achieved with traditional antihypertensive drugs, the BP-lowering effects of GLP-1 RAs can translate into meaningful cardiovascular risk reduction at the population level and provide additive BP benefit when used alongside conventional therapies. Among individuals with hypertension, GLP-1 RAs are generally well tolerated, although small increases in heart rate and potential interactions with volume-regulating medications may warrant clinical attention.

**Conclusion:**

As newer GLP-based therapies continue to emerge, a clearer understanding of their effects on BP may inform more integrated approaches to cardiometabolic care.

## Introduction

Hypertension commonly coexists with obesity and type 2 diabetes mellitus (T2DM), forming a high-risk cardiometabolic triad.^1^ Excess adiposity is a major contributor to BP elevation, with estimates suggesting that obesity causes 65%-78% of primary hypertension cases.^2^ In parallel, hypertension is highly prevalent among individuals with T2DM, affecting approximately 78% of patients.^3^ The coexistence of obesity and T2DM markedly amplifies cardiovascular risk and contributes to poor BP control in clinical practice. Glucagon-like peptide-1 receptor agonists (GLP-1 RAs), initially developed for glycemic control among people with T2DM, have gained increasing attention for their broader metabolic and cardiovascular benefits. In addition to lowering blood glucose and promoting weight loss, GLP-1 RAs have been shown to reduce BP across a range of randomized controlled trials (RCTs).^4^^,^^5^ Given their expanding use in populations with obesity, T2DM, and cardiovascular disease, a deeper understanding of their impact on BP is increasingly relevant. This review outlines the mechanistic pathways through which GLP-1 RAs influence BP, summarizes RCT evidence across relevant populations, and discusses implications for clinical practice and future research.

## Mechanistic pathways

**Figure 1.**
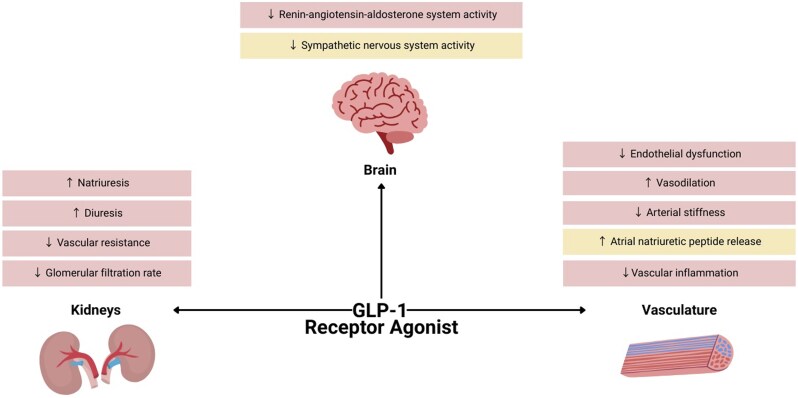
Proposed mechanistic pathways underlying the direct BP-lowering effects of GLP-1 receptor agonists, independent of weight loss. GLP-1 receptor agonists exert renal, vascular, and central effects that converge to reduce BP. Pink boxes denote mechanisms supported by clinical studies, and yellow boxes denote mechanisms primarily demonstrated in preclinical studies.

GLP-1 receptors are expressed in multiple tissues relevant to cardiovascular and renal physiology, including the kidneys, vasculature, and nervous system.^6^ Within the kidneys, GLP-1 receptors are predominantly located in the proximal tubule, where they modulate sodium reabsorption and contribute to natriuresis.^7^ In the vasculature, GLP-1 receptors have been identified on both endothelial and smooth muscle cells, where they influence vascular tone and inflammatory signaling.^8^^,^^9^ In the brain and peripheral nervous system, GLP-1 receptor expression is concentrated in regions such as the hypothalamus and brainstem,^10^ supporting a role in autonomic regulation. This widespread receptor distribution provides a mechanistic basis for the multifaceted effects of GLP-1 RAs on BP reduction (Figure 1).

### Renal effects and natriuresis

GLP-1 RAs promote natriuresis, likely through inhibition of the sodium-hydrogen exchanger 3 in the renal proximal tubule.^7^^,^^11^^,^^12^ This effect has been demonstrated in both animal and human models and contributes to mild reductions in plasma volume, lowering cardiac preload, vascular resistance, and systemic BP.^7^^,^^13–15^ Notably, the natriuretic response appears to occur independently of glycemic control.^15^ There is also evidence that GLP-1 RAs improve renal hemodynamics by increasing renal blood flow and reducing glomerular hyperfiltration,^15^ an effect particularly relevant in patients with early diabetic kidney disease.^16^ These changes may lower intraglomerular pressure and confer renal protection, with secondary benefits on BP control.

### Vascular endothelial function and inflammation

Activation of vascular GLP-1 receptors has been linked to enhanced nitric oxide bioavailability, reduced oxidative stress, and attenuation of endothelial dysfunction, all of which contribute to improved vasodilation and reduced arterial stiffness.^8^^,^^17^ GLP-1 RAs may also stimulate atrial natriuretic peptide release, a cardiac hormone with vasodilatory properties, although available evidence is mainly preclinical.^18^ In addition, GLP-1 RAs exert anti-inflammatory properties, including downregulation of pro-inflammatory cytokines (eg, IL-6, TNF-α), decreased adhesion molecule expression, and suppression of monocyte infiltration into the vascular wall.^19^ By improving both vascular structure and function, GLP-1 RAs may contribute to more sustained BP reductions over time.

### Neurohormonal modulation

GLP-1 RAs may modulate BP by influencing neurohormonal activity and autonomic tone. Preclinical studies suggest that GLP-1 receptor activation in the brainstem can suppress sympathetic outflow and lower circulating norepinephrine levels, potentially reducing systemic vascular resistance.^20^^,^^21^ GLP-1 receptors are also expressed in peripheral structures such as the carotid body, where they may dampen chemoreflex-driven sympathetic activation,^22^^,^^23^ although this reduced activation has yet to be confirmed in human studies. GLP-1 RAs also interact with the renin-angiotensin-aldosterone system (RAAS) but consistent changes in angiotensin II or aldosterone levels have not been demonstrated.^13^ Overall, neurohormonal mechanisms may contribute BP lowering effects, particularly in patients with heightened sympathetic tone, but their clinical relevance is less established than renal or vascular pathways. Collectively, these three pathways suggest that GLP-1 RAs may exert direct effects on BP regulation independent of weight loss.

### Weight loss

Weight loss is a well-established strategy for lowering BP, with a 5%-10% decrease in body weight linked to SBP and DBP reductions of approximately 5 and 4 mm Hg, respectively.^24^ These effects result from several interrelated mechanisms, including improvements in insulin sensitivity, reductions in sympathetic activity, decreased plasma volume, and favorable changes in vascular function, many of which overlap with the direct pathways described above.^25^^,^^26^ In head-to-head RCTs, GLP-1 RAs that induce greater weight loss are also associated with larger BP reductions,^27^^,^^28^ supporting a meaningful contribution of weight-mediated mechanisms to their antihypertensive effect. Variability in weight loss response across individuals has been observed in RCTs, which may contribute to heterogeneity in BP effects.^4^^,^^29^ More recently, dual GLP-1/glucagon and triple GLP-1/glucagon/glucose-dependent insulinotropic polypeptide (GIP) RAs have been developed to achieve more pronounced weight loss and have demonstrated larger BP reductions than single GLP-1 RAs in early-phase RCTs.^30^^,^^31^ These findings support weight loss as a key driver of the BP-lowering effects observed with GLP-1-based therapies.

## Clinical evidence

### Evidence among individuals with diabetes

Major cardiovascular outcome trials of GLP-1 RAs in individuals with T2DM have consistently demonstrated modest reductions in SBP (Table 1).^32–39^ These trials primarily enrolled participants at high cardiovascular risk, most of whom had established hypertension and were receiving background antihypertensive therapy. In this setting, GLP-1 RAs were administered at lower doses indicated for glycemic control rather than weight management. Correspondingly, weight loss across these trials was more limited, ranging from 0.7 to 4.3 kg. BP was generally assessed as a secondary or exploratory endpoint. Reported reductions in SBP ranged from approximately 1 to 3 mm Hg. For example, liraglutide in LEADER reduced SBP by a mean of 1.2 mm Hg,^33^ while injectable and oral semaglutide in SUSTAIN-6 and PIONEER-6, respectively, achieved mean reductions of 2.6 mm Hg.^34^^,^^37^ Smaller effects were noted with lixisenatide in ELIXA (−0.8 mm Hg), possibly reflecting its shorter-acting profile and the acute coronary syndrome population studied.^32^ Changes in DBP were generally minimal and inconsistent across trials. These findings suggest that GLP-1 RAs confer modest BP-lowering effects as adjuncts to standard antihypertensive therapy in individuals with T2DM at elevated cardiovascular risk.

**Table 1.** Summary of BP changes in cardiovascular outcomes trials assessing GLP-1 RAs among individuals with type 2 diabetes.^a^

Trial	Year	Sample size	Population	GLP-1 RA	**Dose** ^b^	**Duration (years, median)** ^c^	BP change (mm Hg)		Weight loss (kg)
							Systolic	Diastolic	
**ELIXA^32^**	2015	6068	Type 2 diabetes and acute coronary syndrome	Lixisenatide	20 μg daily	2.1	−0.8	NR	−0.7
**LEADER^33^**	2016	9340	Type 2 diabetes and high cardiovascular risk	Liraglutide	1.8 mg daily	3.8	−1.2	−0.6	−2.3
**SUSTAIN-6^34^**	2016	3297	Type 2 diabetes	Semaglutide	1 mg weekly	2.1	−2.6	0.1	−4.3
**EXSCEL^35^**	2017	14,752	Type 2 diabetes	Exenatide	2 mg weekly	3.2	−1.6	0.3	−1.3
**HARMONY-O^36^**	2018	9463	Type 2 diabetes and cardiovascular disease	Albiglutide	50 mg weekly	1.6	−0.7	NR	−0.8
**PIONEER-6^37^**	2019	3183	Type 2 diabetes and high cardiovascular risk	Semaglutide (oral)	14 mg daily	1.3	−2.6	0.7	−3.4
**REWIND^38^**	2019	9901	Type 2 diabetes with prior cardiovascular event or risk factors	Dulaglutide	1.5 mg weekly	5.4	−1.7	0.1	−1.5
**AMPLITUDE-O^39^**	2021	4076	Type 2 diabetes and high cardiovascular risk	Efpeglenatide	4 or 6 mg weekly	1.8	−1.5	−0.6	−2.6

Abbreviations: GLP-1 RA, glucagon-like peptide-1 receptor agonist; NR, not reported.

a Values represent placebo-adjusted mean differences at maximum follow-up.

b Dose listed correspond to those tested in cardiovascular outcome trials and may differ from approved weight loss regimens.

c Duration presented as median follow-up length in years.

### Evidence among individuals overweight/obesity without diabetes

Larger BP reductions have been observed in RCTs evaluating GLP-1 RAs for weight management among individuals with overweight or obesity without diabetes (Table 2).^27^^,^^29^^,^^40–43^ These trials enrolled participants with fewer comorbidities, lower background use of antihypertensives, and, in many cases, undiagnosed or untreated hypertension. GLP-1 RAs were administered at higher doses than earlier T2DM-focused RCTs to optimize weight loss, leading to mean reductions ranging from 5.3 to 21.2 kg. In the STEP program, semaglutide consistently reduced BP, with mean SBP reductions ranging from 4 to 9 mm Hg and mean DBP reductions from 2 to 6 mm Hg.^27^^,^^29^^,^^40^^,^^43^ Similarly, SURMOUNT-1, which evaluated tirzepatide, a dual GLP-1/GIP RA, reported mean reductions of 6.4 mm Hg SBP and 3.6 mm Hg DBP at the highest dose.^41^ In comparison, SELECT was a cardiovascular outcome trial that enrolled individuals with obesity and established cardiovascular disease but without diabetes. Although it shared similarities with weight management trials in design and dosing, BP reductions were more modest (−3.3 mm Hg SBP, −0.6 mm Hg DBP), likely reflecting the high baseline cardiovascular risk and widespread use of antihypertensive drugs in the study population.^42^ Collectively, these obesity-focused RCTs highlight the potential of GLP-1-based therapies to deliver meaningful BP improvements alongside weight loss in individuals without diabetes.

**Table 2.** Summary of BP changes in randomized controlled trials assessing GLP-1 RAs among individuals with overweight/obesity.^a^

Trial	Year	Sample size	Population	GLP-1 RA	**Dose** ^b^	**Duration** ^c^ **(weeks)**	BP change (mm Hg)		Weight loss (kg)
							Systolic	Diastolic	
**STEP-1^29^**	2021	1961	Overweight/Obesity	Semaglutide	2.4 mg weekly	68	−5.1	−2.4	−12.7
**STEP-3^43^**	2021	611	Overweight/Obesity	Semaglutide	2.4 mg weekly	68	−3.9	−2.2	−10.6
**STEP-5^40^**	2022	304	Overweight/Obesity	Semaglutide	2.4 mg weekly	104	−4.2	−3.7	−12.9
**STEP-8^27^**	2022	338	Overweight/Obesity	Liraglutide	3 mg daily	68	−6.1	−1.2	−5.3
				Semaglutide	2.4 mg weekly		−8.9	−5.7	−13.8
**SURMOUNT-1^41^**	2022	2539	Overweight/Obesity	Tirzepatide	15 mg weekly	72	−6.4	−3.6	−21.2
**SELECT^42^**	2023	17,604	Overweight/Obesity and cardiovascular disease	Semaglutide	2.4 mg weekly	143 (median)	−3.3	−0.6	−8.3

Abbreviations: GLP-1 RA, glucagon-like peptide-1 receptor agonist.

a Values represent placebo-adjusted mean differences at maximum follow-up.

b Doses listed correspond to approved weight management doses for liraglutide and semaglutide and highest trial dose for tirzepatide.

c Duration presented as trial follow-up in weeks unless otherwise noted.

### Weight-dependent versus weight-independent BP effects

Some early diabetes RCTs suggested notable weight-independent effects of liraglutide on BP. In LEAD-2 and LEAD-5, investigators reported that SBP reductions occurred prior to substantial weight loss and may not have been fully explained by changes in body weight.^44^^,^^45^ A subsequent review of the LEAD program similarly concluded that liraglutide was associated with meaningful BP reductions despite minimal weight loss in some trials (Table 3).^44–50^ For example, in LEAD-4, liraglutide reduced SBP by a mean of 6.7 mm Hg despite a mean weight loss of 1.0 kg only.^49^ In contrast to these earlier observations, contemporary obesity RCTs with larger sample sizes and longer follow-up have enabled more detailed analyses of the extent to which BP reductions are mediated by weight loss versus alternate pathways.^51–53^

**Table 3.** Summary of weight and BP changes in trials assessing liraglutide in type 2 diabetes.^a^

**Trial** ^b^	Description	Change in body weight (kg)	Change in SBP (mm Hg)
**LEAD-1^47^**			
**Liraglutide 0.6 mg**	26-week study of 1041 patients who previously received ≥1 oral antidiabetic drug; all study treatments were in combination with glimepiride 2-4 mg/day	0.7	−0.9
**Liraglutide 1.2 mg**		0.3	−2.6
**Liraglutide 1.8 mg**		−0.2	−2.8
** Placebo**		−0.1	−2.3
**LEAD-2^44^**			
**Liraglutide 0.6 mg**	26-week study of 1091 patients who previously received ≥1 oral antidiabetic drug; all study treatments were in combination with metformin 1 g 2×/day	−1.8	−0.6
**Liraglutide 1.2 mg**		−2.6	−2.8
**Liraglutide 1.8 mg**		−2.8	−2.3
**Placebo**		−1.5	−1.8
**LEAD-3^48^**			
**Liraglutide 1.2 mg**	52-week, active-controlled, study of 746 patients previously on diet/exercise program or receiving oral antidiabetic drug	−2.1	−2.1
**Liraglutide 1.8 mg**		−2.5	−3.6
**LEAD-4^49^**			
**Liraglutide 1.2 mg**	26-week study of 533 patients who previously received ≥1 oral antidiabetic drug; all study treatments were in combination with metformin 1 g 2×/day and rosiglitazone 4 mg 2×/day	−1.0	−6.7
**Liraglutide 1.8 mg**		−2.0	−5.6
**Placebo**		0.6	−1.1
**LEAD-5^45^**			
**Liraglutide 1.8 mg**	26-week study of 581 patients who previously received ≥1 oral antidiabetic drug; all study treatments were in combination with metformin 1 g 2×/day and glimepiride 4 mg/day	−1.8	−4.0
**Placebo**		−0.4	−1.4
**LEAD-6^50^**			
**Liraglutide 1.8 mg**	26-week, open-label, active-controlled study of 464 patients who previously received metformin and/or sulfonylurea; all study treatments were in combination with metformin and/or sulfonylurea	−3.2	−2.5

a Table is adapted from Montanya et al.^46^ Values represent mean changes from baseline at trial end.

b LEAD-1 to LEAD-6 correspond to phase III trials of liraglutide; All trials were conducted in adults with type 2 diabetes with varying background therapy.

**Figure 2.**
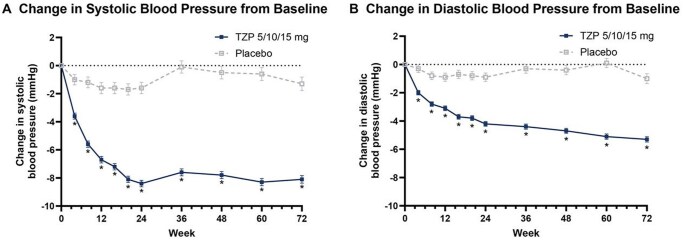
Effects of tirzepatide on BP in SURMOUNT-1 over 72 weeks. (A) SBP change from baseline, (B) DBP change from baseline. Reprinted with permission from Krumholz et al, *BMJ Heart* 2024;110:290-300.

In SURMOUNT-1, analyses of BP changes over 72 week showed that clinically significant reductions were evident as early as week 9, preceding the full extent of weight loss achieved by tirzepatide in the trial (Figure 2).^51^^,^^53^^,^^54^ Mediation analyses estimated that approximately 68% of the SBP reduction and 71% of DBP reduction were attributable to weight loss,^53^ suggesting that nearly one-third of the effect arose from alternate pathways. In contrast, mediation analyses from an individual participant data meta-analysis (IPDMA) pooling STEP-1, STEP-3, and STEP-4 estimated that 89% of the SBP reduction with semaglutide was mediated by weight loss.^52^ Changes in SBP were strongly correlated with changes in body weight, and participants who did not lose weight experienced minimal BP change. Nonetheless, since mediation analyses rely on strong assumptions, these findings should be interpreted with caution, and causal inferences regarding the exact contribution of weight-independent pathways cannot be definitively established.^55^

### Comparison to traditional antihypertensive therapies and clinical relevance

**Figure 3.**
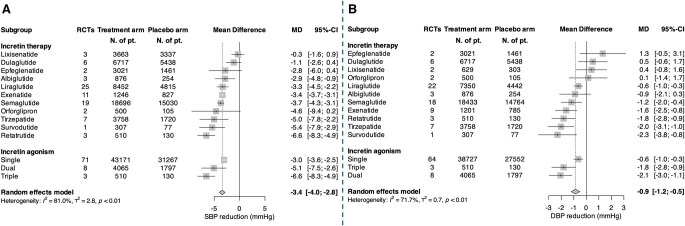
Forest plot of randomized controlled trials evaluating the effect of GLP-1 receptor agonists and co-agonists on (A) systolic and (B) diastolic blood pressure among overweight/obese adults, with or without diabetes. Mean differences (mmHg) with 95% confidence intervals were pooled using random-effects models. Negative values indicate greater blood pressure reduction with GLP-1 receptor agonists or co-agonists compared to placebo. Reprinted with permission from Basile et al.^60^

Traditional antihypertensive medications, such as angiotensin-converting enzyme (ACE) inhibitors, thiazide diuretics, or calcium channel blockers, typically reduce SBP by 7 to 11 mm Hg (Table 4).^56–59^ In comparison, large meta-analyses of GLP-1 RA trials support a pooled SBP reduction of approximately 3 mm Hg (Figure 3, Table 5),^4^^,^^5^^,^^60^^,^^61^ with greater effects observed with dual or triple agonists (SBP reductions of −5.1 and −6.6 mm Hg, respectively).^60^ Although modest at the individual level, a 5 mm Hg SBP reduction has been associated with about a 10% lower risk of major cardiovascular events, including 13% reductions in both stroke and heart failure, 8% in ischemic heart disease, and 5% in cardiovascular death, making these effects important at a population level.^62^

**Table 4.** Comparative effects of GLP-1 RAs and traditional antihypertensive drug classes on BP.^a^

Drug class	BP change (mm Hg)		Additional effects	Notes
	Systolic	Diastolic		
**GLP-1 RAs**	−3.4 (−4.0 to −2.8)	−0.9 (−1.2 to −0.5)	Weight loss, improved glycemia, cardiovascular protection	Greater effect with dual/triple co-agonists
**ACE inhibitors**	−6.8 (−7.7 to −5.9)	−4.1 (−4.7 to −3.6)	Renal protection	First-line in diabetes and chronic kidney disease
**Angiotensin II receptor blockers**	−8.5 (−9.3 to −7.8)	−5.5 (−6.0 to −5.0)	Renal protection	Similar to ACE inhibitor; fewer cough/angioedema
**Thiazide diuretics**	−10.8 (−12.4 to −9.2)	−5.6 (−6.6 to −4.6)	Volume reduction	May worsen glucose and lipids
**Calcium channel blockers**	−9.5 (−10.6 to −8.4)	−6.5 (−7.3 to −5.6)	Anti-anginal	Useful in elderly, isolated systolic hypertension
**Beta blockers**	−8.9 (−10.0 to −7.8)	−6.8 (−7.8 to −5.8)	Heart rate reduction	Less effective for stroke protection

Abbreviations: ACE, angiotensin-converting enzyme; ARB, angiotensin II receptor blocker; GLP-1 RA, glucagon-like peptide-1 receptor agonist.

a BP effects are derived from large meta-analyses of randomized controlled trials.^4^^,60^ Values represent placebo-adjusted mean differences (95% confidence interval).

**Table 5.** Summary of large meta-analyses assessing the effects of GLP-1 RAs and co-agonists on BP.^a^

Author, Year	Population	Trials (*n*)/Participants (*N*)	**Interventions** ^b^	BP change (mm Hg)	
				Systolic	Diastolic
**In Chou et al, 2025^61^**	Type 2 diabetes and/or obesity	75/114,352	Retatrutide	−7.0 (−10.5 to −3.5)	−1.7 (−2.6 to −0.9)
			Tirzepatide	−5.2 (−6.9 to −3.5)	−1.4 (−3.1 to 0.4)
			Semaglutide	−3.4 (−4.7 to −2.0)	−0.8 (−1.4 to −0.2)
			Liraglutide	−2.7 (−3.9 to −1.5)	−0.5 (−1.1 to 0.1)
			Efpeglenatide	−3.5 (−8.0 to 0.9)	0.7 (−1.6 to 3.0)
			Orforglipron	−2.5 (−4.5 to −0.5)	−0.1 (−0.9 to 0.7)
			Albiglutide	−1.8 (−5.2 to 1.7)	−0.9 (−3.1 to 1.3)
			Dulaglutide	−1.7 (−3.2 to −0.1)	−0.4 (−0.9 to 0.2)
			Exenatide	−1.5 (−3.1 to 0.1)	−0.6 (−1.3 to 0.2)
**Basile et al, 2025^60^**	Type 2 diabetes and/or obesity		Retatrutide	−6.6 (−8.3 to −4.9)	−1.8 (−2.8 to −0.9)
		85/90,977	Survodutide	−5.4 (−7.9 to −2.9)	−2.3 (−3.8 to −0.8)
			Tirzepatide	−5.0 (−7.8 to −2.2)	−2.0 (−3.1 to −1.0)
			Semaglutide	−3.7 (−4.3 to −3.1)	−1.2 (−2.0 to −0.4)
			Liraglutide	−3.3 (−4.5 to −2.2)	−0.6 (−1.0 to −0.3)
			Efpeglenatide	−2.8 (−6.0 to 0.4)	1.3 (−0.5 to 3.1)
			Orforglipron	−4.6 (−9.4 to 0.2)	0.1 (−1.4 to 1.7)
			Albiglutide	−2.9 (−4.8 to −0.9)_	−0.9 (−2.1 to 0.3)
			Dulaglutide	−1.1 (−2.6 to 0.4)	0.5 (−0.6 to 1.7)
			Exenatide	−3.4 (−3.7 to −3.1)	−1.6 (−2.5 to −0.8)
			Lixisenatide	−0.3 (−1.6 to 0.9)	0.4 (−0.8 to 1.6)
			**Pooled** ^c^	**−3.4 (−4.0 to −2.8)**	**−0.9 (−1.2 to −0.5)**
**Rivera et al, 2024^5^**	Type 2 diabetes and/or obesity	63/53,072	Semaglutide	−3.4 (−4.2 to −2.6)	−0.7 (−1.5 to −0.1)
			Liraglutide	−2.6 (−3.5 to −1.7)	−0.2 (−0.6 to 0.1)
			Dulaglutide	−1.5 (−2.2 to −0.7)	0.3 (−0.1 to 0.6)
			Exenatide	−3.4 (−3.6 to −3.1)	−0.9 (−1.8 to −0.1)
**Wong et al, 2025^4^**	Overweight/Obesity	30/37,072	Semaglutide	−4.0 (−4.9 to −3.1)	−1.5 (−2.1 to −0.9)
			Liraglutide	−2.7 (−3.3 to −2.2)	−0.7 (−1.1 to −0.3)
			Exenatide	−2.3 (−4.0 to −0.6)	−0.5 (−3.4 to 2.4)
			**Pooled**	**−3.4 (−4.0 to −2.8)**	**−1.1 (−1.5 to −0.7)**

Abbreviations: GIP, glucose-dependent insulinotropic polypeptide; GLP-1 RA, glucagon-like peptide-1 receptor agonist.

a Values represent pooled placebo-adjusted mean differences (95% confidence interval).

b Retatrutide is a triple agonist (GLP-1/GIP/glucagon). Tirzepatide and survodutide are dual agonists (GLP-1/GIP and GLP-1/glucagon, respectively). All other agents are selective GLP-1 RAs.

c Bold values indicate the pooled overall mean difference for all agents combined within each meta-analysis.

## Subgroup analyses and special populations

The impact of GLP-1 RAs on BP has been examined across a range of clinical subgroups and high-risk populations (Table 6).

**Table 6.** BP effects of GLP-1 RAs across clinical subgroups and high-risk populations.

Subgroup	**Findings** ^a^	Remaining gaps
**Age/Sex**	Effects consistent across strata in SURMOUNT-1 and meta-analyses^4^ ^,^ ^53^	Limited data in postmenopausal women
**Race/Ethnicity**	Similar effects across groups in SUSTAIN program^63^	Underrepresentation of non-White participants
**Baseline BP**	No strong effect modification in STEP, SURMOUNT and SELECT^52^ ^,^ ^53^ ^,^ ^65^ ^,^ ^66^	Unclear in resistant hypertension
**Resistant hypertension**	Higher rates of antihypertensive de-escalation in IPDMA of STEP-1, 3, and 4^52^	No dedicated RCTs
**Chronic kidney disease**	Modest SBP reduction and renal protection in AWARD-7, REWIND and FLOW^67–69^	Sparse data without diabetes
**Heart failure**	Neutral in HFrEF^75^; modest benefit in HFpEF^76^ ^,^ ^77^	Need phenotype-specific studies
**Obstructive sleep apnea**	BP reductions parallel improvements in apnea severity^78^ ^,^ ^79^	Mechanistic versus weight-mediated contributions unclear

Abbreviations: GLP-1 RA, glucagon-like peptide-1 receptor agonist; HFrEF, heart failure with reduced ejection fraction; HFpEF, heart failure with preserved ejection fraction; IPDMA, individual participant data meta-analysis; RCT, randomized controlled trial.

a Findings are based on post hoc analyses or subgroup-specific reports from RCTs unless otherwise indicated.

### Age and sex

The BP-lowering effects of GLP-1 RAs appear to be largely consistent across age- and sex-defined subpopulations. In stratified analyses of SURMOUNT-1, tirzepatide lowered SBP and DBP similarly across age and sex subgroups, with no suggestion of a differential response.^53^ These findings are supported by a 2025 meta-analysis of 30 RCTs including over 37,000 individuals with overweight or obesity.^4^ In this analysis, meta-regression showed no significant association between BP reduction and mean baseline age or the proportion of male participants.^4^ However, data specific to postmenopausal women, who may have distinct vascular and metabolic profiles, remain limited. Further research is needed to evaluate whether hormonal status or age-related changes in endothelial function influence the response to GLP-1 RAs in this population.

### Race and ethnicity

Relatively few RCTs have conducted formal subgroup analyses by race or ethnicity, and available data are limited by underrepresentation of non-White participants. A post hoc analysis of the SUSTAIN program suggested that semaglutide’s BP effects were largely similar across racial and ethnic groups. In SUSTAIN-6, a small increase in SBP (+1.9 mm Hg) was observed among Black participants receiving the 0.5 mg dose of semaglutide, although this subgroup was small (*n *= 54) and the effect was not seen at the 1.0 mg dose.^63^ Given known disparities in hypertension prevalence, control rates, and treatment response across racial groups,^64^ further research is needed to assess whether GLP-1 RAs provide comparable benefits across diverse populations. Dedicated RCTs or real-world studies that specifically enroll Black, Hispanic, Indigenous, or other underrepresented populations may help fill this gap.

### Baseline BP level

Effect modification by baseline BP appears limited across RCTs. In STEP-1, semaglutide reduced both SBP and DBP compared with placebo. Exploratory analyses found no meaningful differences in effect between participants with baseline BPs above versus below the cohort median baseline BP or between those with controlled versus uncontrolled hypertension.^65^ Similarly, in SURMOUNT-1, tirzepatide reduced SBP across baseline thresholds of 120, 130, and 140 mm Hg, with no statistically significant interaction by strata.^53^ In SELECT, semaglutide reduced SBP across all baseline categories, with reductions ranging from −2.9 mm Hg in participants without hypertension to −3.7 mm Hg in those with stage 2 hypertension, without a marked increase in effect among higher baseline BP groups.^66^ An IPDMA pooling STEP-1, 3 and 4 confirmed consistent SBP reductions across multiple hypertension definitions (diagnosed hypertension, baseline SBP >130 mm Hg, baseline SBP >140 mm Hg, resistant hypertension [RHTN]),^52^ although reductions were less pronounced and uncertain in the RHTN subgroup. Overall, current evidence does not suggest a substantially greater BP-lowering effect among individuals with higher baseline BP.

### Resistant hypertension

Resistant hypertension remains a particularly challenging and high-risk group, in which patients fail to achieve BP control despite the concurrent use of ≥3 antihypertensive drugs. In the STEP-1, 3, and 4 IPDMA, semaglutide was associated with more frequent de-escalation of antihypertensive therapy compared with placebo in the overall cohort (7.1% vs 3.5%).^52^ Notably, the largest relative benefit was seen in the RHTN subgroup. In this subgroup, 27% of participants randomized to semaglutide de-escalated therapy versus 3% for placebo, and 36% no longer met RHTN criteria at study completion versus 21% in the placebo group. Post hoc analyses further suggested that RHTN participants with baseline SBP ≥130 mm Hg experienced a mean SBP reduction of approximately 11 mm Hg compared with placebo, whereas those with SBP <130 mm Hg had a mean SBP increase of approximately 5 mm Hg but similar rates of medication de-escalation.

It is important to note that many cases of RHTN in clinical practice reflect poor adherence rather than true pharmacological resistance. In this context, GLP-1 RAs may offer a practical advantage: Patients with obesity may be more motivated to adhere to a therapy that also promotes weight loss and improves metabolic health, compared with adding a fourth or fifth antihypertensive agent. As such, GLP-1 RAs may not only improve BP control but also reduce the need for multidrug therapy in RHTN, a clinically relevant outcome warranting further investigation in dedicated trials.

### Chronic kidney disease

GLP-1 RAs have been associated with modest SBP reductions in patients with chronic kidney disease (CKD), with evidence derived largely from T2DM trials.^67–69^ In AWARD-7 and REWIND, dulaglutide lowered SBP by approximately 1-2 mm Hg while slowing the decline in estimated glomerular filtration rate and reducing albuminuria among individuals with moderate-to-severe CKD.^67^^,^^68^ In FLOW, semaglutide reduced the risk of major kidney outcomes among individuals with T2DM and established CKD, with concurrent small SBP reductions also observed.^69^ Evidence among individuals with CKD without T2DM remains scarce and no dedicated subgroup analyses from large obesity or cardiovascular outcome trials have reported BP effects in this population. While sodium-glucose cotransporter 2 (SGLT2) inhibitors remain the first-line therapy in most CKD guidelines,^70^ GLP-1 RAs may provide complementary renal and hemodynamic benefits, particularly among patients with obesity or poor glycemic control.^71^

### Heart failure

Evidence for the BP effects of GLP-1 RAs in heart failure is limited and varies by phenotype. In heart failure with reduced ejection fraction (HFrEF), low baseline BP is common due to reduced cardiac output and use of background therapies. In such patients, further BP reduction is generally undesirable. GLP-1 RAs have also been associated with small increases in heart rate (∼2-5 beats per minute),^72–74^ which could be problematic in this population. In the LIVE trial, liraglutide had no meaningful impact on SBP or left ventricular function in HFrEF but increased heart rate by approximately 7 beats per minute.^75^ Nonetheless, a subset of patients with coexisting obesity and hypertension may still derive benefit from modest BP reductions achieved with GLP-1 RAs.

In contrast, in heart failure with preserved ejection fraction (HFpEF), elevated BP is a major driver of disease progression, and targeted pharmacological therapies remain limited. Given the frequent coexistence of HFpEF, obesity, and hypertension, the BP-lowering effects of GLP-1 RAs may be more advantageous in this setting. In the STEP-HFpEF and STEP-HFpEF DM trials, semaglutide resulted in modest reductions in SBP (−2.9 mm Hg) alongside improvements in symptoms, physical function, and weight.^76^ A pooled analysis of the SELECT, FLOW, and STEP-HFpEF trials also demonstrated reduced risk of worsening heart failure events with semaglutide, with consistent effects across baseline SBP defined subgroups (<130 vs ≥130 mm Hg).^77^ Overall, GLP-1 RAs appear more relevant in HFpEF, particularly with concomitant obesity, where BP lowering contributes to broader cardiometabolic benefits rather than a direct heart failure therapy.

### Obstructive sleep apnea

Obstructive sleep apnea (OSA) is strongly linked to hypertension through mechanisms including sympathetic overactivity, intermittent hypoxia, and obesity-related hemodynamic stress. Weight loss is a cornerstone of OSA management, and GLP-1 RAs may offer dual benefits in this population by improving both apnea severity and BP control. In the SCALE Sleep Apnea trial, liraglutide led to a SBP reduction of 4.1 mm Hg, paralleling improvements in the apnea-hypopnea index, among patients with obesity and moderate-to-severe OSA.^78^ More recently, in the SURMOUNT-OSA trials, tirzepatide led to substantial weight loss and improved OSA severity, with BP effects that differed depending on concomitant continuous positive airway pressure use (7.6 mm Hg SBP and 2.8 mm Hg DBP reductions in nonusers versus 3.7 mm Hg and 1.1 mm Hg in users).^79^ While the mechanisms likely involve both weight-dependent and independent effects, dedicated BP-focused trials in OSA populations are necessary to clarify the causal pathways and magnitude of effect.

## Safety considerations

As with any antihypertensive therapy, the use of GLP-1 RAs requires consideration of safety and tolerability (Table 7).

**Table 7.** Safety considerations of GLP-1 RAs relevant to BP regulation.^a^

Concern	Evidence	Clinical implications
**Heart rate elevation**	Increases of 2-5 beats per minute consistently observed, especially with long-acting agents^72–74^	Monitor in patients with CAD or arrhythmias
**Volume depletion**	Mild natriuretic/diuretic effects; rare hypotension in RCTs^45^ ^,^ ^53^ ^,^ ^77^	Monitor in patients with fluid loss or on multiple antihypertensives; caution in frail adults or when combined with SGLT2 inhibitors or diuretics
**Orthostatic hypotension**	Not systematically assessed	Consider BP monitoring on standing in older adults or those with autonomic dysfunction, volume depletion, or complex antihypertensive regimens
**RAAS interaction**	Mechanistic evidence inconsistent^13^ ^,^ ^15^; no adverse signals in RCTs	Safe with ACE inhibitors/ARBs; caution with dual RAAS blockade

Abbreviations: ACE, angiotensin-converting enzyme; ARB, angiotensin II receptor blocker; CAD, coronary artery disease; GLP-1 RA, glucagon-like peptide-1 receptor agonist; RAAS, renin-angiotensin-aldosterone-system; RCT, randomized controlled trial; SGLT2, sodium-glucose cotransporter-2.

a No consistent signal for increased adverse cardiovascular events attributable to these safety concerns has been observed in large cardiovascular outcome trials.

### Heart rate elevation and autonomic effects

One of the most consistent physiological effects observed with GLP-1 RAs is a modest, dose-dependent increase in heart rate, typically ranging from 2 to 5 beats per minute.^72–74^ This chronotropic effect is more pronounced with long-acting agents,^73^ such as liraglutide and semaglutide, and may reflect attenuation of parasympathetic tone.^80^ While the clinical significance of this heart rate increase remains uncertain, it raises theoretical concerns in patients with ischemic heart disease or arrhythmias and may counteract some of the antihypertensive benefit of GLP-1 RAs. Notably, large cardiovascular outcome trials have not demonstrated an increased risk of major adverse cardiovascular events attributable to heart rate changes.^33^^,^^34^^,^^42^ However, longer-term safety data, particularly in high-risk subgroups, are needed to clarify whether sustained heart rate elevation modifies cardiovascular risk over time.

### Volume depletion and drug-drug interactions

While GLP-1 RAs are generally well tolerated and are not associated with hypotension to the extent seen with traditional antihypertensive drugs,^81^ their natriuretic effects may contribute to mild volume depletion.^13^^,^^15^ Although the magnitude of this effect in clinical settings remains unclear, there is a biological rationale to consider an elevated risk of volume-related symptoms in susceptible individuals, such as older adults, those with orthostatic hypotension, or patients on multidrug antihypertensive regimens.^82^ This risk may also be more pronounced when GLP-1 RAs are used in combination with SGLT2 inhibitors or diuretics.^71^ Large RCTs and pooled analyses have not shown a clear signal for increased adverse events related to hypotension or dizziness,^44^^,^^53^^,^^77^ and combination therapy with SGLT2 inhibitors has been well tolerated in dedicated trials such as SUSTAIN-9 and LIRA-ADD2SGLT2i.^83^^,^^84^ Nonetheless, product labeling for GLP-1 RAs advises monitoring volume status, especially in patients with fluid loss or concurrent antihypertensive use.^85^

Limited mechanistic evidence suggests that GLP-1 RAs may interact with the RAAS,^13^^,^^15^ but the clinical relevance of this interaction remains uncertain. Coadministration with ACE inhibitors or angiotensin II receptor blockers (ARB) has not been associated with excess adverse events in RCTs. In routine practice, most patients with hypertension already receive one of these agents and the addition of a GLP-1 RA appears safe. Caution remains warranted among those on multiple RAAS blockers, such as an ACE inhibitor/ARB plus a mineralocorticoid receptor antagonist, where overlapping hemodynamic or electrolyte effects may emerge.

## Clinical implications and practical considerations

### Integration into hypertension management

The consistent, albeit modest, reductions in BP observed with GLP-1 RAs have important implications for cardiometabolic care. By targeting glycemia, weight, and BP simultaneously, GLP-1 RAs offer a pleiotropic benefit that distinguishes them from traditional antihypertensive drugs and positions them as valuable adjuncts in comprehensive risk reduction strategies. The 2025 American Heart Association/American College of Cardiology Blood Pressure Guidelines acknowledge the BP-lowering effects of GLP-1 RAs, particularly among individuals with overweight or obesity without diabetes, based on the STEP and SURMOUNT-1 trials.^86^ While not indicated for BP management, GLP-1 RAs are frequently prescribed to individuals with obesity-related hypertension or metabolic syndrome. These populations often require multidrug regimens to achieve BP targets. In such contexts, the addition of a GLP-1 RA may not only provide additive benefit through hemodynamic and metabolic effects but also support simplification of pharmacotherapy or de-escalation of other antihypertensive agents. As clinical use expands and future RCTs assess BP as a primary outcome, the role of GLP-1 RAs in hypertension management will likely become clearer.

### Patient-level considerations

Clinicians considering GLP-1 RAs for patients with suboptimally controlled hypertension should be mindful of existing antihypertensive therapy. In particular, dose adjustments may be warranted in those taking diuretics or other agents that influence fluid balance to minimize the risk of hypotension or volume depletion. Home BP monitoring during dose escalation may be helpful, particularly for patients with labile BP or those on multidrug regimens. GLP-1 RAs may also be useful in patients with masked or prehypertension who are not yet candidates for conventional antihypertensive therapy. In such cases, particularly among those with obesity or T2DM, initiation of a GLP-1 RA for metabolic reasons may simultaneously delay or prevent the need for a dedicated antihypertensive agent.

### Duration of treatment

The optimal duration of GLP-1 RA use for patients with obesity and coexisting hypertension remains unclear. BP reductions with GLP-1 RAs typically emerge early and persist with continued therapy, paralleling improvements in weight and metabolic parameters. However, discontinuation often leads to weight regain and loss of cardiometabolic benefits,^42^^,^^87^ suggesting that, as with traditional antihypertensive drugs, sustained BP control may require ongoing use. In some cases, improved BP may permit de-escalation of other antihypertensive agents, although this may raise the risk of BP rebound if the GLP-1 RA is subsequently withdrawn. Clinicians should set expectations accordingly and individualize treatment duration based on therapeutic response, tolerability, and long-term management goals.

### Adherence to GLP-1 therapy

Adherence is a major challenge in hypertension management, with suboptimal medication use contributing to poor BP control. Similar concerns apply to GLP-1 RAs, where long-term adherence is essential to maintain both metabolic and hemodynamic benefits. In RCTs, adherence is typically high, supported by close follow-up and structured care.^40–43^ However, real-world studies indicate substantial discontinuation, with 30%-50% of patients stopping treatment within one year.^88^^,^^89^ Common reasons include gastrointestinal side effects, injection burden, high cost, and limited insurance coverage.^90^ Supporting adherence through gradual dose titration, proactive side effect management, patient education, and regular follow-up may help sustain treatment benefits over time, although broader system-level efforts will be needed to improve long-term affordability and access.

## Future research directions

### Trials with BP as a primary outcome

Most data on GLP-1 RAs come from trials where BP was a secondary or exploratory outcome, with assessment methods ranging from clinic-based readings to self-monitored values. Heterogeneity in baseline antihypertensive drug use, trial duration, GLP-1 RA dosing, and participant characteristics further limits the comparability and interpretation of BP effects across RCTs. Future trials that designate BP as a prespecified primary endpoint, using standardized and preferably automated measurement protocols, could better define the magnitude and consistency of the antihypertensive effect. Stratification by baseline BP, body mass index, antihypertensive drug use, and diabetes status may help identify whether certain patient subgroups derive greater benefit. Dedicated studies comparing GLP-1 RAs to conventional antihypertensives or evaluating their additive effects when combined with existing regimens will help clarify their role in treatment algorithms. Randomized crossover trials or studies incorporating ambulatory BP monitoring could also distinguish acute hemodynamic effects from longer-term vascular adaptations.

### Mechanistic and precision medicine studies

In addition to clinical outcomes, mechanistic studies using biomarkers, vascular imaging, and renal function assessments may improve our understanding of how GLP-1 RAs lower BP. Identifying whether specific effects, such as nitric oxide enhancement, sympathetic inhibition, or natriuresis are more pronounced in certain individuals could pave the way for a more personalized approach to therapy. For instance, patients with obesity-related hypertension or salt-sensitive BP may be more likely to benefit from GLP-1 RAs than those with isolated systolic hypertension driven by arterial stiffness.

### Real-world evidence

Real-world data can complement trial evidence by evaluating how GLP-1 RAs influence BP in routine clinical practice. Observational studies using linked electronic health records, prescription databases, or ambulatory BP monitoring can assess treatment patterns, adherence, and comparative effectiveness and safety across more heterogeneous and underrepresented populations. These findings will help bridge the gap between trial data and everyday care, informing future guideline development and implementation strategies as GLP-1 RAs are increasingly used across broader populations and indications.

## Conclusions

GLP-1 RAs produce modest but consistent reductions in BP, typically in the range of 2-5 mm Hg systolic, with the largest effects seen in agents associated with greater weight loss. Although modest at an individual level, decreases of this magnitude can translate into meaningful reductions in the occurrence of major cardiovascular events at the population level. These benefits likely arise through a combination of weight-mediated and weight-independent mechanisms, including natriuresis, improved vascular function, and autonomic modulation. While BP reduction is not their primary indication, GLP-1 RAs are increasingly prescribed to patients with T2DM, obesity, and metabolic syndrome, which are populations in which hypertension is common and often difficult to control. In these contexts, their pleiotropic effects offer a unique opportunity to address multiple cardiometabolic risk factors within a single agent. Ongoing research will be important to clarify the optimal treatment duration, the potential for integration within existing antihypertensive treatment regimens, and the long-term impact of GLP-1 RAs on hypertension-related outcomes.

## Data Availability

No new data were generated or analysed in support of this research.
